# Identification of plant leaf phosphorus content at different growth stages based on hyperspectral reflectance

**DOI:** 10.1186/s12870-020-02807-4

**Published:** 2021-01-07

**Authors:** Anna Siedliska, Piotr Baranowski, Joanna Pastuszka-Woźniak, Monika Zubik, Jaromir Krzyszczak

**Affiliations:** 1 Institute of Agrophysics, Polish Academy of Sciences, ul. Doświadczalna 4, 20-290 Lublin, Poland; 2 Department of Biophysics, Institute of Physics, Maria Curie-Skłodowska University, 20-031 Lublin, Poland

**Keywords:** Hyperspectral imaging, Supervised classification, Phosphorus fertilization, Precision agriculture

## Abstract

**Background:**

Modern agriculture strives to sustainably manage fertilizer for both economic and environmental reasons. The monitoring of any nutritional (phosphorus, nitrogen, potassium) deficiency in growing plants is a challenge for precision farming technology. A study was carried out on three species of popular crops, celery (*Apium graveolens* L., cv. Neon), sugar beet (*Beta vulgaris* L., cv. Tapir) and strawberry (*Fragaria × ananassa* Duchesne, cv. Honeoye), fertilized with four different doses of phosphorus (P) to deliver data for non-invasive detection of P content.

**Results:**

Data obtained via biochemical analysis of the chlorophyll and carotenoid contents in plant material showed that the strongest effect of P availability for plants was in the diverse total chlorophyll content in sugar beet and celery compared to that in strawberry, in which P affects a variety of carotenoid contents in leaves. The measurements performed using hyperspectral imaging, obtained in several different stages of plant development, were applied in a supervised classification experiment. A machine learning algorithm (Backpropagation Neural Network, Random Forest, Naive Bayes and Support Vector Machine) was developed to classify plants from four variants of P fertilization. The lowest prediction accuracy was obtained for the earliest measured stage of plant development. Statistical analyses showed correlations between leaf biochemical constituents, phosphorus fertilization and the mass of the leaf/roots of the plants.

**Conclusions:**

Obtained results demonstrate that hyperspectral imaging combined with artificial intelligence methods has potential for non-invasive detection of non-homogenous phosphorus fertilization on crop levels.

**Supplementary Information:**

The online version contains supplementary material available at 10.1186/s12870-020-02807-4.

## Background

Phosphorus (P) is an essential macronutrient that greatly influences root development, plant growth and crop productivity [1]. Moreover, P has a significant function in various metabolic processes in plants, such as protein formation, photosynthesis, cell division, respiration, energy storage and nutrient movement within the plant, and is an integral constituent of nucleic acids, phospholipids, and coenzymes activating amino acid production [2]. P has been found to be one of the most important minerals for celery (*Aqium graveolens*) growth, quality and yield. It was responsible for increasing the total above-ground mass, marketable trimmed yield and yield of larger grade sizes [3]. Phosphorus also plays an important role in sugar beet development because it is essential for root yield and sugar assimilation [4, 5].

Inappropriate P fertilizer management is dangerous for both plants and the environment and generates additional costs. Fertilizers are commonly applied according to farmer experience, which can easily lead to over- or misapplication, resulting in soil quality degradation, reduction in crop yields, contamination of the environment, deterioration of water quality, eutrophication, and loss of biodiversity [6]. Therefore, adequate phosphorus fertilization plays a key role in precision agriculture. Currently, information about P status in plants is obtained by visual inspection or chemical analyses, which are costly, time consuming and laborious. Moreover, these methods are destructive, precluding their usage for the continuous monitoring of P content and thus P resources in the field during plant growth. As a result, alternative and efficient methods of P content monitoring are needed in plants.

It is well known that phosphorus deficiency in plants disturbs the production of chlorophyll, causing leaf chlorosis [7, 8]. Prolonged P deficiency may further result in the accumulation of anthocyanins, consequently leading to purple discolouration on the leaf surface [9, 10]. The above-mentioned changes alter the spectral reflectance characteristics of leaves or canopies and enable the application of spectral reflectance methods, such as leaf colour charts and chlorophyll metres, for the nondestructive estimation of phosphorus status [11, 12]; however, most of these methods focus on individual leaves. Hyperspectral imaging, which combines spectroscopy with imaging methods, allows collection of canopy images and delivers representative reflectance data that are useful for the determination of plant phosphorus status in the field. In recent years, this technique has been effectively employed for various crops to estimate biophysical parameters, such as leaf area index [13, 14], leaf and fruit pigment content [15–21], biomass [22] as well as detection of diseases and fungal infections [23–26]. Several studies have been reported on the spectral changes related to leaf water content [27, 28], chlorophyll content [29, 30] and macronutrient content, e.g., nitrogen [31, 32] and potassium [33].

The objective of the automatic detection of nutritional deficiencies is to identify the visual symptoms that characterize such deficiencies. Most previous studies have focused on estimating the contents of biochemical constituents in leaves as the response to phosphorus deficiency of a single plant species, such as citrus leaves [34, 35], rice [36], wheat [37], and oilseed rape [6, 38]. Christensen et al. [39] indicated that P content could be predicted with 74% accuracy based on the spectral canopy reflectance. Similarly, high accuracy (correlation coefficient of 0.710) has been obtained for the determination of P content in oilseed rape leaves using eight wavelengths selected from the visible and near infrared (VIS-NIR) spectrum [6]. Mahajan et al. [40] proposed the two-band (combination of 1080 nm and 1460 nm wavelengths) vegetation index for the prediction of P content in wheat. Most of these studies have focused on the direct prediction of P content based on reflectance indices combining a few spectral bands or on indirect detection by predicting the content of a related substance (e.g., chlorophyll content). Until now, few investigations have been dedicated to analysing the temporal dynamics of leaf morphology and colour under different P treatments covering longer periods of plant growth and development and multiple bands of visible/infrared spectrum [41, 42].

It is evident from previous studies that the monitoring of P status in different crops using hyperspectral systems is possible; however, more attention should be paid to properly characterize plant spectral response to varied P fertilization, including the key stages of growth. Moreover, to our knowledge, there is still insufficient effort dedicated to the classification of nutritional anomalies in root vegetables (such as wild celery - *Apium graveolens L.*). These limitations have become the prerequisites for undertaking research to develop robust and more specific algorithms for predicting P status in plants (including root vegetables) at different development stages and fertilization doses.

The aim of the present study was to develop a discrimination model for monitoring the dynamics of plant phosphorus (P) status across the different developmental stages of wild celery, strawberry and sugar beet crops under different P fertilizations using hyperspectral reflectance measurements. The three species selected for this study are very popular in temperate climatic zones due to the economic importance (sugar beet is the main source of sugar in many countries), as well as their nutritional value and taste (celery and strawberry).

## Results and discussion

### Reference data of chlorophyll and macronutrient content

**Fig. 1:**
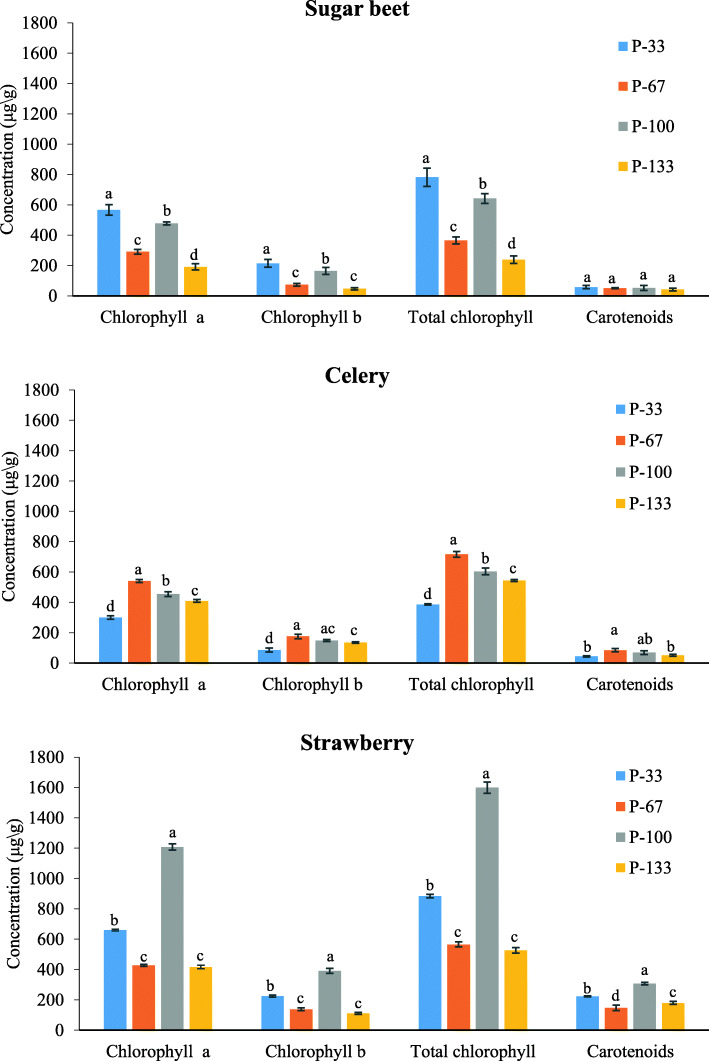
Measured content of Chlorophyll a, Chlorophyll b, total Chlorophyll and Carotenoids in sugar beet (**a**), celery (**b**) and strawberry (**c**) plants under different P applications. Bars followed by the same letter do not differ statistically by Tukey’s test at *p*=0.05

Different P treatments caused major variations in pigment content in all the studied plants. Figure 1 illustrates the contents of Chlorophyll a_,_ Chlorophyll b_,_ Total chlorophyll and Carotenoids in sugar beet, celery and strawberry plants in response to different P fertilizations. The results show that, in the case of sugar beet and strawberry plants, exceeding the recommended dose of phosphorus in the nutrient solution (yellow bars in Fig. 1 depicting a 33% increase of P) caused a decrease in the chlorophyll content, which refers to all the measured kinds of chlorophyll (Chlorophyll a_,_ Chlorophyll b and Total chlorophyll). The same trend was observed in celery plants, but to a smaller extent. For the three studied species, the maximum chlorophyll concentrations were recorded for various P doses: for sugar beet, the dose was under 33% of the recommended dose, for celery, under 67% of the recommended dose and, for strawberry, under the recommended dose. These differences speak to the varying impacts of P on the chlorophyll activity of various species. The deficiency or excessive application of P into the growing pots caused very high decreases in chlorophyll and carotenoid concentrations only in strawberry leaves compared to the control group (those with the recommended dose), which can be related to leaf chlorosis. This result indicates a high sensitivity of strawberry plants to imbalanced phosphorus dosing, in agreement with observations of Trejo-Téllez and Gómez-Merino [43], who noticed a considerable decrease of chlorophyll content in P-deficient strawberry leaves, which became uniformly yellow under P stress. Additionally, Estrada-Ortiz et al. [44] confirmed a strong relationship between P content in strawberry plants and the accumulation chlorophylls in its leaves. Moreover, these authors indicated that excess P application causes a decrease in the contents of photosynthetic pigments and also influences serious soil and environmental degradation. Figure 1 shows that the concentrations of chlorophyll a in the studied species were much higher than the concentrations of chlorophyll b. This result is not in agreement with Costa et al. [45], who observed that Chlorophyll b concentrations were higher than Chlorophyll a in 2 cultivars of strawberry under varied lightening conditions. However, it was previously indicated that the relationship between chlorophyll a and b depends on many factors, including source of light, shading, ambient conditions of plant growth [46–48] and the specific role of these two pigments in plant physico-chemistry. Chlorophyll a is responsible for the collection of photons and plays an essential role in photosynthesis, while chlorophyll b additionally participates in the transference of light radioactive energy [49, 50].

In celery leaves, the chlorophyll concentration (for all 3 types of chlorophyll) at the lowest P treatment (P-33) was much lower than in other P treatments, indicating that such low P supply has a stress effect on celery. For celery leaves, the highest values of chlorophyll concentration occurred for the variant P-67, not P-100, which speaks to the overestimation of the recommended P dose in the fertilizer in this case. The same was noticed for the carotenoid content in celery leaves (the highest value was noticed for the P-67 variant). It was also observed in celery plants that the highest dose of P in fertilizer (P-133 variant) did not lead to such high decreases in chlorophyll a, b or total concentrations, as was the case for the lowest fertilizer dose (variant P-33), which suggests that celery is more sensitive to the scarcity of P than to its excess.

**Fig. 2:**
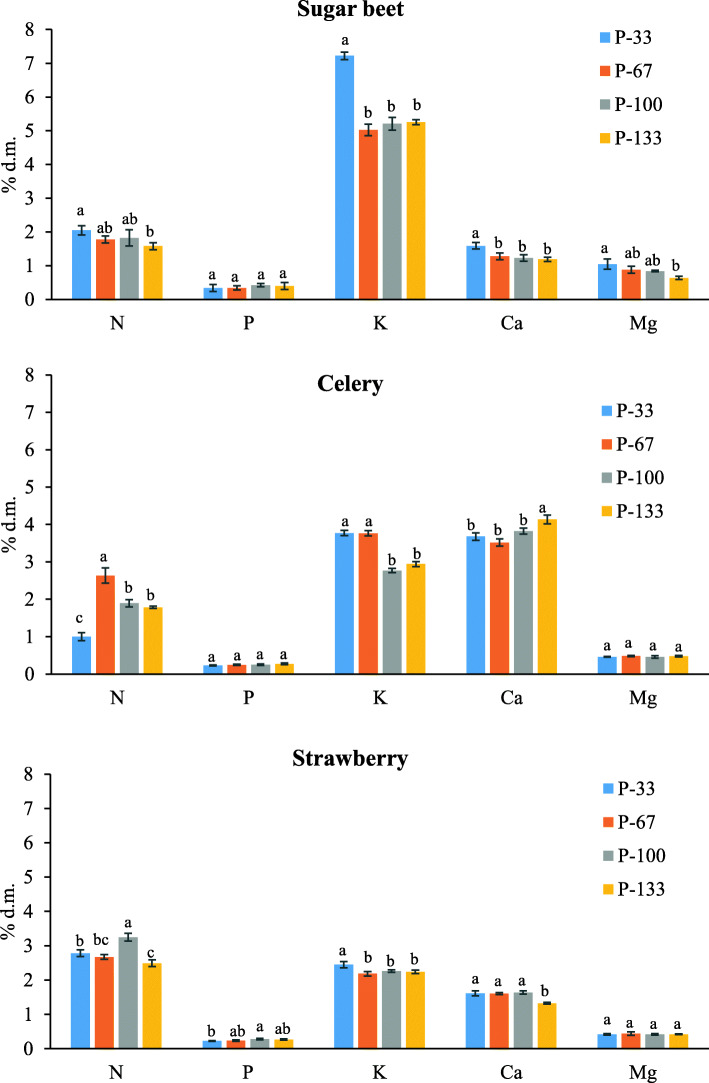
Effect of phosphorus treatment on N, P, K, Ca and Mg in leaf samples determined by traditional methods. Bars followed by the same letter are not significantly different according to Tukey’s test (*p*< 0.05)

The total N, P, K, Mg and Ca contents, measured by reference methods at 49, 51 and 45 DAT for celery, sugar beet and strawberry plants, are shown in Fig. 2. Generally, considerable and statistically significant differences in macronutrient contents were observed between various P treatments in the studied species. However, neither foliar P concentrations in the sugar beet and celery plants nor Mg concentrations in celery and strawberry were significantly affected by P treatment. It was difficult to find strict tendency in macronutrient content changes in the leaves of the three studied species with changing P fertilization. For example, in sugar beet and celery, the lowest dose of P in fertilizer (P-33) led to the highest values of N, which could be due to specific interactions between nutrient elements in the substrate, as explained in research conducted by Y. Li et al. [3]. Similarly, the lowest dose of P in fertilizer (P-33) was reflected in the highest concentrations of K for each of the species. The highest contents of Ca were observed in celery leaves; however, increasing trends with rising P concentrations in the treatments were not confirmed in sugar beet or strawberry. These results confirm the complicated relationships between the contents of macronutrients in leaves and P treatments in the soil, which was also suggested in other sources [3, 51, 52].

### Correlation between leaf biochemical constituents, phosphorus fertilization and mass of the leaf/roots of the plants

**Fig. 3:**
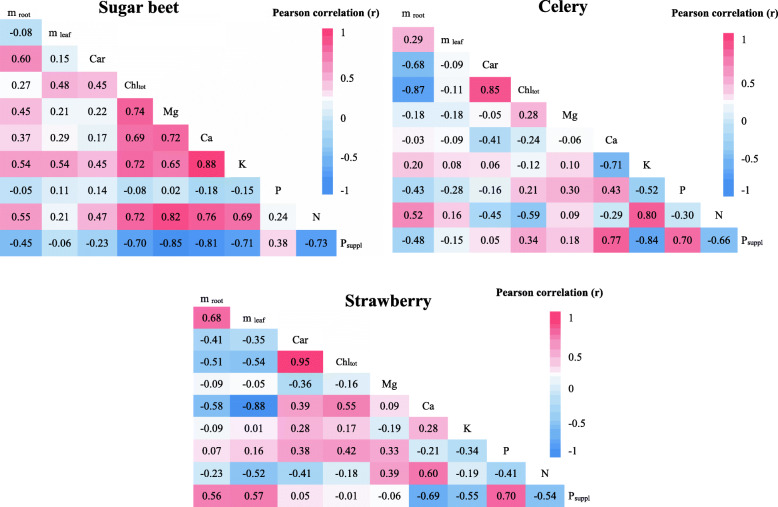
Correlations between carotenoids (Car), chlorophyll (Chl_tot_), magnesium (Mg), calcium (Ca), potassium (K), phosphorus (P), nitrogen (N) content in leaf, leaf mass (mleaf), root mass (mroot) and phosphorus supplementation (P_suppl_)

Pearson correlation coefficients (PCC) between the leaf macronutrients (N, P, K, Ca, Mg), total chlorophyll (Chl_tot_), carotenoids (Car), phosphorus fertilization level (P_suppl)_, mass of the leaf (m_leaf_) and mass of root (m_root_) for the studied species are presented in Fig. 3 as correlation matrices. The leaf pigments and nutritional elements were evaluated through laboratory analysis at the end of the experiment. All plants showed a negative correlation between the level of P supply and the concentration of nitrogen (N) and potassium (K) macronutrients. The results obtained for celery showed a strong positive correlation (PCC=0.77) between the applied dosed of phosphorus fertilizer and the calcium content in plant leaves, whereas the other plants indicated a negative correlation (PCC=-0.81 for sugar beet and PCC=-0.69 for strawberry). Earlier reports also indicated a strong phosphorus fertilization effect on other macronutrient accumulation in plants [53, 54].

A negative and highly significant correlation was observed between the level of P supplementation and chlorophyll concentration in sugar beet, while the other plants showed non-significant correlations. The applied fertilization had no strong effect on the concentrations of carotenoids in plant leaves. Phosphorus fertilization was positively correlated with the concentration of this element in plant leaves, especially celery (PCC=0.70) and strawberry plants (PCC=0.70). Sugar beet showed a smaller correlation between the level of P supplementation and P content in plant leaves. This dependence is consistent with some observations in previous studies indicating that P fertilization increases the phosphate content of sugar beet roots [55]. Most of the plant nutritional elements were highly correlated with each other. Numerous significant correlations existed between nutrients in sugar beet, especially between K and Ca (PCC=0.88) and N and Mg (PCC=0.82). The strong correlations between chlorophyll content and carotenoids were observed for celery (PCC=0.85) and strawberry (PCC=0.95) plants. This is because chlorophylls and carotenoids are co-varying in nature (as a components of photosynthetic antenna complexes) and statistically dependent, as observed in previous studies [16, 56].

Negative correlations between P supplementation in soil and mass of the plant roots suggest that P deficiency promotes a reduction in the mass and length of roots in root vegetables and causes the reduction in yield. In the case of strawberry, this correlation was positive. The concentration of chlorophylls and carotenoids in the above-ground parts of the tested plants significantly affected the mass of their roots. Sugar beet had a positive correlation between the carotenoid content and root mass (PCC = 0.6), whereas a strong negative correlation was observed between the concentration of chlorophyll and carotenoid content in leaves and root mass for celery plants.

### Spectral features of plants

**Fig. 4:**
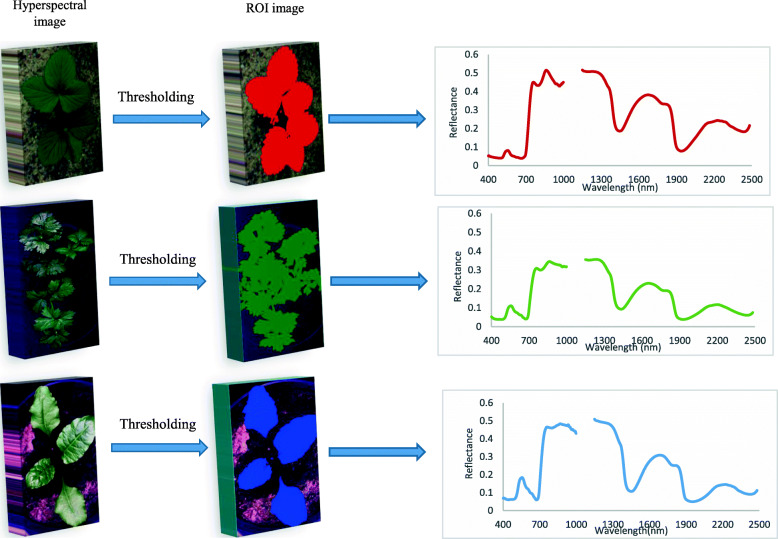
General scheme of the procedure to generate spectral characteristics from hyperspectral images of the three studied plants

**Fig. 5:**
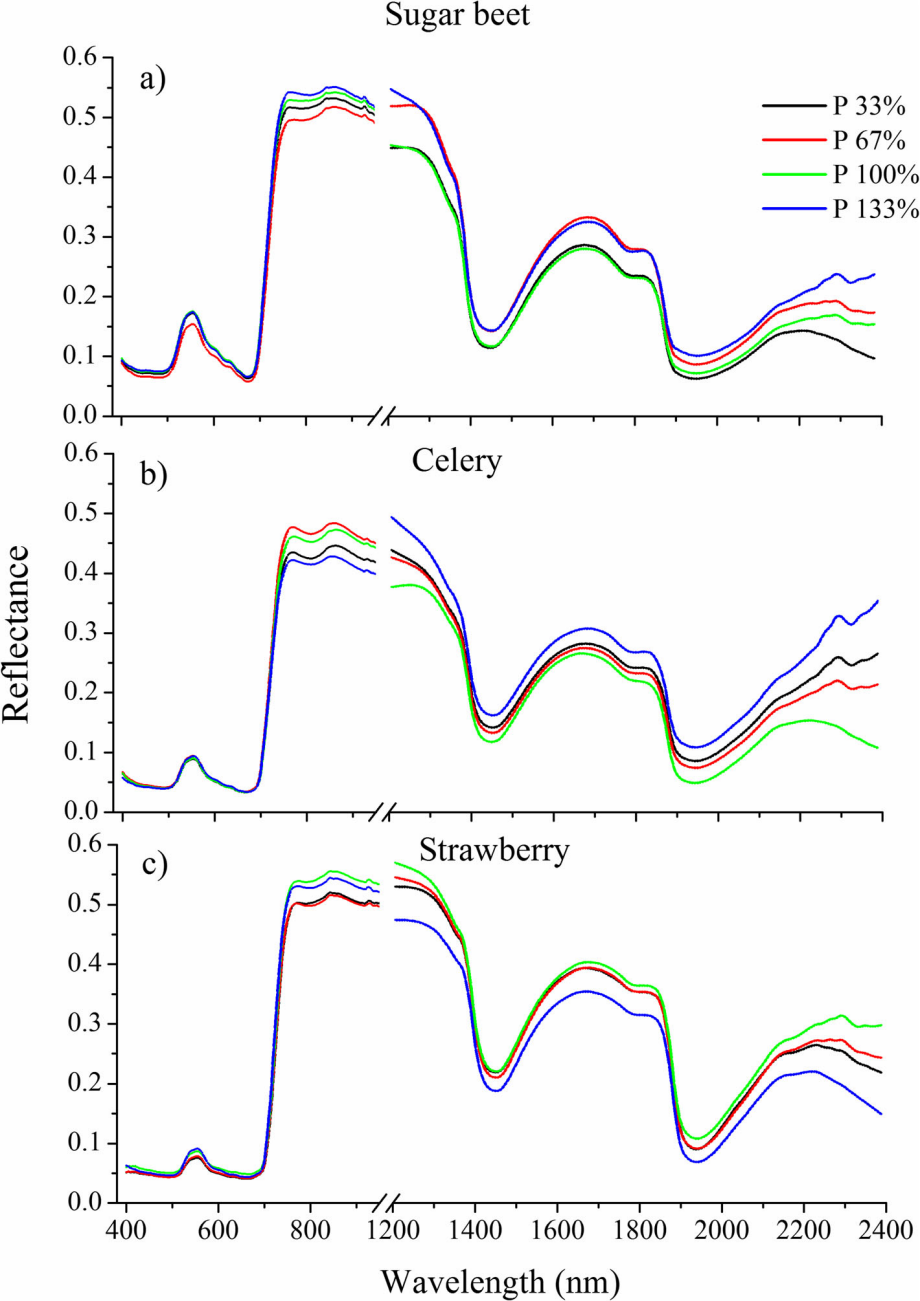
Average reflectance spectra of sugar beet (**a**), celery (**b**) and strawberry plants (**c**) grown under different phosphorus (P) fertilization rates obtained for third development stage. Each line correspond to the spectral characteristics averaged for four plants from each variants of the experiment

Figure 4 represents the general scheme of the procedure to obtain spectral characteristics from the leaf surfaces of the three studied plants. The average reflectance spectra of ROIs, covering the spectral range of 400–2500 nm for leaf samples of the three studied species of plants with different P treatments and for five development stages, are shown in Fig. 5. The spectral curves of the leaves of the three studied species exhibited similar shapes, although differences are visible between the spectra belonging to specific variants. In the visible spectral region, a characteristic peak was observed at 550 nm with some differences between variants, especially in sugar beet and strawberry. This peak is characteristic of chlorophyll absorption. In the region of rapid change in the reflectance of vegetation in the near infrared range of 650–750 nm of the electromagnetic spectrum (so called red edge), high increases of reflectance occur, which enabled us to distinguish differences between some variants of the experiments. The highest differentiation between the spectral curves of the plants belonging to specific variants was observed in the range of 750–1300 nm, in which reflectance patterns are strongly connected with the internal cellular structure of plants [57]. Unfortunately, in this range, there was a break (discontinuity) in the registered reflected radiation, which is connected to low sensitivity of the two spectral cameras used in the part of this range. Because of this, the raw spectra of the leaves in this range were not good at distinguishing between variants. Another part of the spectrum that seems to be appropriate for distinguishing differences between variants is absorption at approximately 1400 and 1950 nm, which are highly related to the absorption by water. The results presented in Fig. 5 indicate quantitative relationships between the amount of reflected light and P treatment at the succeeding growing stages. In plants of all three species, the highest changes in reflectance values were observed in the SWIR region (2200–2400 nm). It suggests that the SWIR region is useful for distinguishing levels of P fertilization. Wavelengths in the SWIR region are mainly associated with light absorption by proteins, nitrogen, cellulose, starch and sugar. It is known that P plays an important role in protein synthesis, which may explain these differences, as suggested by Knox et al. [58].

### Effective wavelength selection

**Fig. 6:**
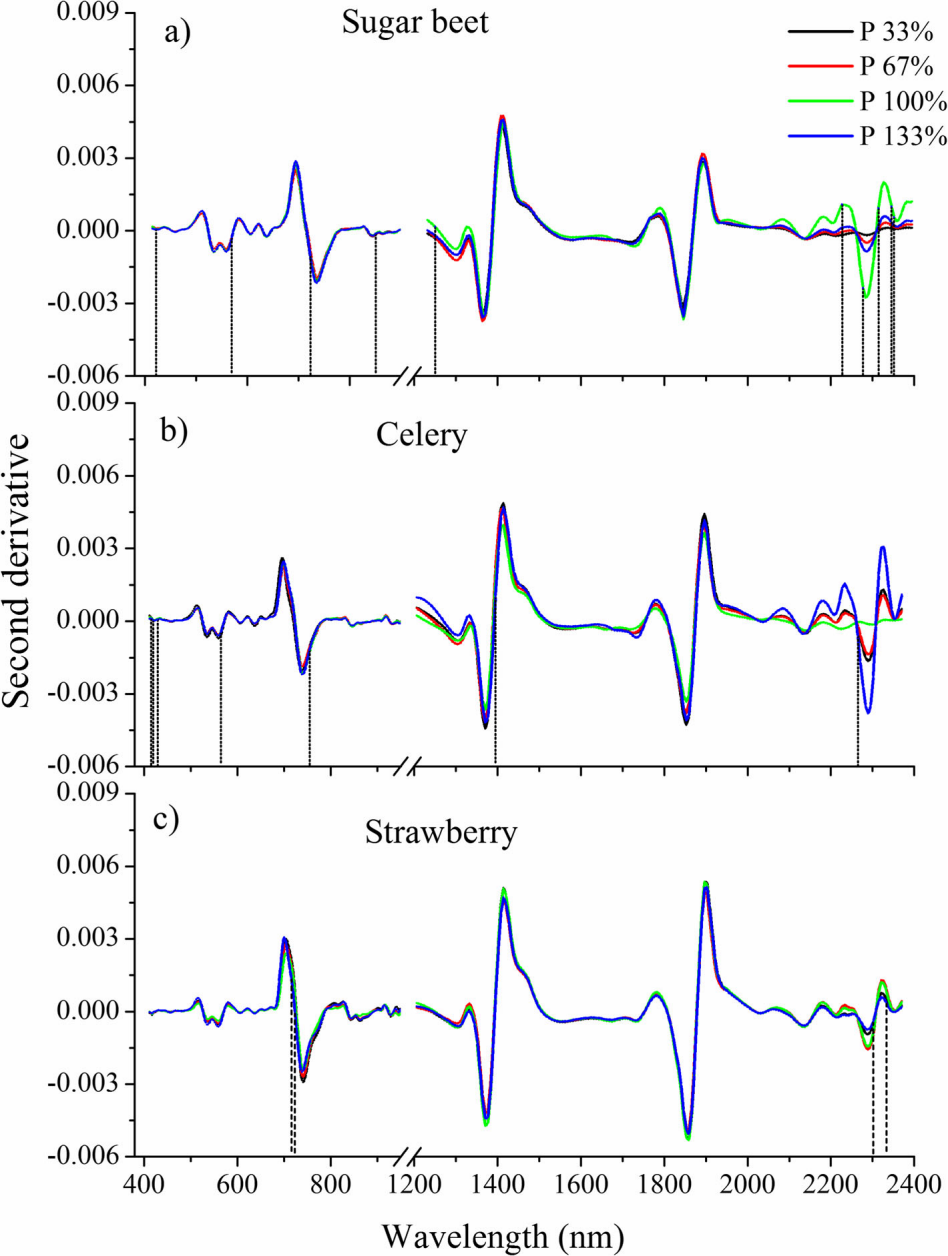
Second derivative transformed spectra of sugar beet (**a**), celery (**b**) and strawberry plants (**c**) grown under different phosphorus (P) fertilization rates obtained for third development stage

To reduce the high dimensionality of the extracted spectral data and to make the classification models more robust, the most appropriate wavelengths that give the highest discrimination among different levels of P-treatment were selected based on 2nd derivative transformation of raw spectra. The 2nd derivative averaged spectra are shown in Fig. 6. Based on the second derivative transformation of the original spectra and by applying the CFS algorithm with greedy stepwise selection method, 10, 7 and 4 wavelengths were selected for classification according to the P treatment of sugar beet, celery and strawberry plants, respectively (Table 1). The wavelengths used to distinguish between levels of P fertilization were localized in the blue spectral band (400–480 nm), NIR (760–900 nm) and SWIR (1000–2500 nm) regions of the spectrum. In all studied plants, the level of P supply did not significantly affect the reflectance in the green (500–560 nm) region. In the case of strawberry plants, the wavelengths from the red region (715 and 723 nm) and SWIR region (2301 and 2332 nm) had particular importance for the separation of the levels of P treatment. The wavelengths in the red and far-red regions of the electromagnetic spectrum (723, 754, 715 and 723 nm) in plants are mainly associated with the absorption of Chlorophyll a. It was shown that varied P rates cause changes in the concentration of Chlorophyll a in plant leaves (Fig. 1).

**Table 1:** Wavelengths selected based on the second derivative transformed spectra and CFS algorithm with greedy-stepwise selection methods

Plant species	Number of selected wavelengths	Selected wavelengths [nm]
Sugar beet	10	422, 569, 723, 850, 1250, 2227, 2276, 2314, 2345, 2351
Celery	7	414, 419, 429, 564, 754, 1395, 2264
Strawberry	4	715, 723, 2301, 2332

The previous study performed by Osborne et al. [9] also indicated that NIR (730 nm and 930 nm) and blue (440 and 445 nm) regions of the spectrum are useful for the prediction of P concentrations in corn canopy. Differences in the selected wavelengths among the three studied species might be due to differences in plant structure or changes in the chemical concentration.

### Results of discrimination analysis

The prediction accuracies of the models created to distinguish between plants grown under different levels of P treatment at different development stages obtained for the three studied plant species are presented in Table 2. It results from the analysis of the supervised classification algorithms that very similar and relatively high prediction accuracies in the majority of cases (ranging from 40 to 100% for validation sets) were obtained for all four methods of machine learning model creation methods (i.e., backpropagation neural network, random forest, naive Bayes and support vector machines). In all cases, despite very limited numbers of wavelengths selected for the classification (from 4 to 10), the prediction accuracies for training sets were very high in all variants of the experiment. This confirms a good performance of the CFS wavelength selection algorithm and is in agreement with other studies on plant material classification with the use of this algorithm [26, 59]. The performance of the validation sets was considerably lower than that of the training sets, but the accuracy at distinguishing between various levels of P treatment were equal or higher than 80% in 11 variants among 15 variants of species/stages of plant development. This result is very good although difficult to compare with other studies that used different experimental setups and limited numbers of P treatment variants [38, 42]. The lowest percentages of correctly classified instances were obtained for the first stage of plant development; however, with progress in the development of plants, this accuracy was higher. This result comes from the fact that, in the first period of plant development, the changes in leaf spectral properties are considerably minimal between various P treatments and misclassification, especially with one level or higher of P fertilization. Although all four methods of supervised classification model creation were highly effective, the highest overall classification performance was obtained for RF models. The validation results indicated that this model correctly classified more than 70% of all instances in the case of strawberry plants and more than 80% (except the second term) for celery across five development stages. The average accuracy of RF classification for sugar beet was lower compared to other plants (65%). This result might be explained by the specific nutrient requirements of the sugar beet [55, 60] and its lower sensitivity of imbalanced P-fertilization than strawberry and celery plants.

**Table 2:** Model performance on selected wavelengths for classification of the level of P treatment at five developmental stages obtained for the three studied species of plants

Plant species			Sugar beet				Celery				Strawberry			
Model			BNN	LIBSVM	LOG	RF	BNN	LIBSVM	LOG	RF	BNN	LIBSVM	LOG	RF
I	Training set	%	98	84	91	100	98	91	100	100	86	79	68	100
		RMSE	0.12	0.28	0.18	0.15	0.12	0.21	0	0.12	0.19	0.32	0.31	0.1
	Validation set	%	55	45	50	65	75	60	80	80	80	60	55	70
		RMSE	0.44	0.52	0.49	0.35	0.3	0.45	0.32	0.32	0.29	0.45	0.39	0.29
II	Training set	%	98	89	100	100	98	93	95	100	97	97	100	100
		RMSE	0.11	0.24	0	0.11	0.12	0.18	0.17	0.13	0.09	0.11	0	0.05
	Validation set	%	70	70	65	70	55	60	50	55	80	90	80	95
		RMSE	0.37	0.39	0.42	0.29	0.39	0.45	0.48	0.34	0.26	0.22	0.31	0.16
III	Training set	%	95	86	100	100	100	91	100	100	98	77	82	100
		RMSE	0.13	0.26	0	0.12	0.06	0.22	0.01	0.11	0.15	0.34	0.22	0.09
	Validation set	%	65	60	40	75	80	95	55	80	100	85	80	95
		RMSE	0.23	0.45	0.55	0.33	0.29	0.16	0.47	0.28	0.17	0.27	0.24	0.15
IV	Training set	%	100	93	100	100	100	97	100	100	100	100	100	100
		RMSE	0.07	0.19	0	0.11	0.05	0.11	0	0	0.02	0	0	0.01
	Validation set	%	55	85	55	90	80	75	65	85	100	100	95	100
		RMSE	0.38	0.27	0.47	0.27	0.27	0.35	0.41	0.27	0.03	0	0.14	0.05
V	Training set	%	87	86	100	100	100	95	100	100	91	86	100	100
		RMSE	0.21	0.26	0	0.13	0.03	0.15	0	0	0.2	0.26	0	0.11
	Validation set	%	60	45	65	70	95	95	90	95	65	50	80	80
		RMSE	0.37	0.52	0.41	0.35	0.15	0.16	0.22	0.16	0.34	0.5	0.32	0.3

**Fig. 7:**
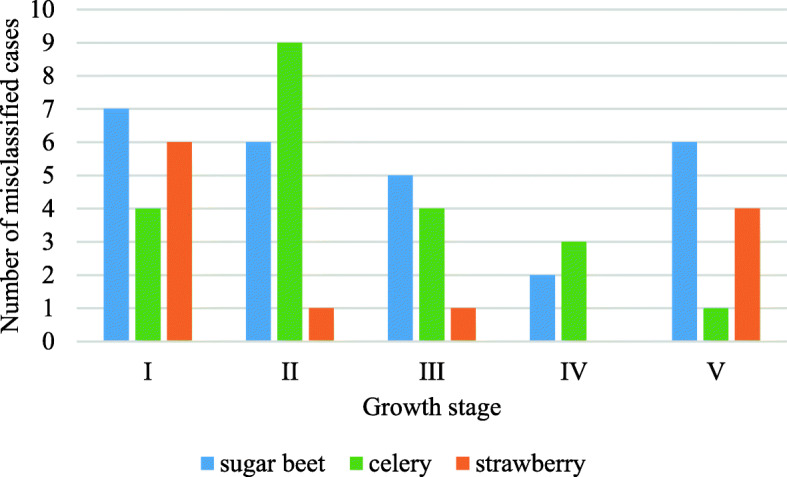
Numbers of misclassified cases in a validation dataset for random forest (RF) models of P content in plant treatment for 5 stages of plant growth and 3 studied species

To assess the performance of the analysed models for specific P levels in five developmental stages, confusion matrices were created, which enabled us to identify misclassification percentages for analysed variants of the experiment. The summary of this analysis is presented in Table 3 for RF models, which gave the best overall results in the performed experiments. The grey cells in this table represent variants with 100% accuracy (all cases classified correctly), yellow cells show misclassified variants in which misclassification refers to one level up or down with respect to the analysed P fertilization level (e.g., P-33 level classified as P-67 level), and red cells indicate misclassification higher than one P fertilization level (e.g., variant P-133 classified as P-33). The confusion matrices for all models divided to 5 growth stages are presented in Table S1 in Supplement 1. For each developmental stage, the percentages of misclassified cases are given, and it is possible to see how misclassification occurred (second column in this table indicates the analysed variants, and separate rows show with which variants they were misclassified and what percent of misclassification occurred). From this table, 100% accuracy was achieved (all cases classified correctly) for 26 variants of the experiment (P level vs development stage), misclassification was one level up or down with respect to the analysed P fertilization level in 27 variants, and misclassification was higher than one P fertilization level in only 13 variants. In the majority of misclassified variants (26), improperly classified cases reached only 20%, there were only 9 variants with misclassified cases of 40%, 3 variants with misclassified cases of 60% and 1 variant with misclassified cases of 80%. Table 3 also shows that there were only 6 variants for which two different levels of P treatment were assigned for a given level, five of which occurred for the first and second plant developmental stages. In Fig. 7, the numbers of misclassified cases in the validation dataset for random forest (RF) models of P content in plant treatment for 5 stages of plant growth and 3 studied species are presented, and these are based on the confusion matrices presented in Table S1 in Supplement 1. This figure shows that the highest number of misclassified cases for sugar beet and strawberry occurred during the first stage of plant growth, whereas this occurred during the second stage of plant growth for celery. This confirms that, in the early stages of plant growth, spectral properties of the affected plant leaves do not always distinguish differences in P content. Despite this, the overall classification performance of the chosen models (and especially RF models) was very good.

**Table 3:**
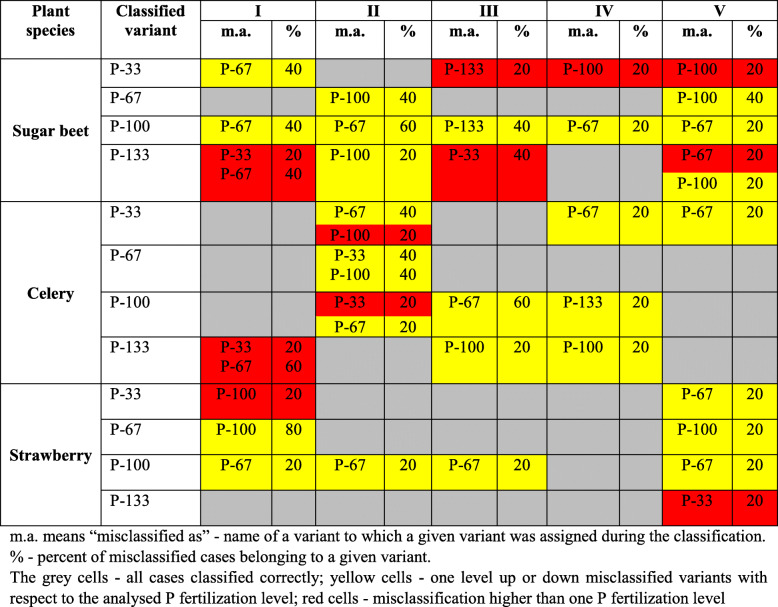
Summary of confusion matrices created for the random forest (RF) models of the phosphorous content in treatments for the three studied species (sugar beet, celery and strawberry) at five stages of plant growth

*m.a* means “misclassified as” - name of a variant to which a given variant was assigned during the classification

% - percent of misclassified cases belonging to a given variant

The grey cells - all cases classified correctly; yellow cells - one level up or down misclassified variants with respect to the analysed P fertilization level; red cells - misclassification higher than one P fertilization level

## Conclusions

There is high potential of hyperspectral screening for controlling adequate phosphorous nutrition of cultivated plants, which is vital for creating proper conditions of their production and responses to environmental factors. The experiment conducted on sugar beet, celery and strawberry indicated that it is possible for these species to distinguish, with high accuracy, the differences in phosphorous nutrition using machine learning modelling on the basis of second derivatives of the reflectance spectra in the spectral region of 450–2500 nm. Moreover, it elaborated that procedures provide a chance to distinguish different levels of phosphorus fertilization at different developmental stages of plants. Phosphorus deficiency can be accurately classified at an early plant developmental stage, especially for celery and strawberry; however, the classification accuracy increases during plant growth.

The results have several practical implications. First, they can be applied to the fast and non-destructive analysis of phosphorous availability for the examined three species as an element of precision farming. In particular, the detection of insufficiencies in P content is possible with multispectral scanners installed on tractor platforms supported with GPS systems, through implementing selected spectral bands into available sensors, and through direct use of the elaborated procedures of data analysis and supervised classification. The method enables us to pin-point areas in the cultivated fields or even individual plants requiring attention. In this context, it is especially important that elaborated models concern different stages of plant growth, which is an additional advantage of such screening. The second possible application of the study is its implementation to unmanned aerial vehicles that are capable of distant analysis of large areas at a time. However, some additional technical aspects should be considered in such systems, such as irregular lightening of the analysed scene, the occurrence of strong vibrations, and controlling flight trajectories.

The authors are aware that further studies are needed to explore the impact of soil type on accumulated phosphorous in plants, interactions between phosphorous status in plants frequently undergoing abiotic and biotic stresses and their representation in reflectance spectra and chemometric analysis. Such additional studies will strengthen the performance of the elaborated method in detecting phosphorous status in plants.

## Methods

### Plant material

The same number of plants of three species was used for the experiment: 64 celery (*Apium graveolens* L., cv. Neon), 64 sugar beet (*Beta vulgaris* L., cv. Tapir) and 64 strawberry (*Fragaria × ananassa* Duchesne, cv. Honeoye). The seeds of celery (produced by SEMO) and sugar beet (produced by SESVanderHave) were purchased commercially, while the strawberry seedlings with qualification certificate were obtained from Licensed Strawberry Nursey (Niewczas Krystyna & Józef, Wielowicz 31, 89–412 Sośno, Poland). On March 2018, seeds of celery (*Apium graveolens* L., cv. Neon) and sugar beet (*Beta vulgaris* L., cv. Tapir) were sown in plastic pots containing peat. After germination, seedlings of similar sizes were transplanted to pots (one seedling per pot) containing sand. The seedlings of strawberry plants were directly placed in pots with sand. The plants were grown in the greenhouse under natural sunlight supplemented white LED light at light intensity of 320 μmol m^− 2^ s^− 1^ using a photoperiod of day/night set to 12/12 h, with temperature ranging from 20 °C to 22 °C during the months of March–June and September and from 24 °C to 26 °C during the months of July–August.

### Treatments

In this experiment, the plants from each species were divided equally into four groups of 16 plants each and were subjected to four different phosphorus rates, which were applied to the pots to stimulate different nutrient levels in plant leaves to test the hyperspectral imaging system. P fertilizer in the form of superphosphate (P_2_O_5_) was applied to the pots directly before seedling in various quantities to obtain different fertilization schemes named P-33%, P-67%, P-100%, and P-133%. In the variant P-100%, which was the control group for the whole experiment, the dose recommended in the literature for these types of varieties was used [61–63], namely 1.2 g P per pot (40 mg/kg of the soil). Other variants were treated with 1/3, 2/3, and 4/3 of this value. After initial differentiation, each plant was irrigated with 100 ml of the treatment solution every two days for 60 days. The nutrient solution contained 33.3 mg/l of N, 13.3 mg/l of P and 50 mg/l of K applied as NH_4_NO_3_, Ca(H_2_PO_4_)_2_ and KNO_3_, respectively. The concentrations of micronutrients were B 0.28, Fe 2.4, Mn 1.0, Zn 0.35, and Mo 0.05 mg/l, which were applied as a commercial fertilizer Micro Plus (produced by Intermag, Olkusz, Poland). After 60 days, a constant level of the soil water content was maintained in pots corresponding to the field capacity of water. To do so, the field capacity of water for the used soil was determined for selected soil samples under the soil water potential of 15,596 J·m^− 3^ (pF equal to 2.2) in the Richard’s chambers (Soilmoisture Equipment Corp., Santa Barbara, CA, USA). At that stage, the plants were watered using tap water, and the appropriate soil water content was controlled using a weighting method.

### Hyperspectral imaging system

Spectral data were recorded by a laboratory hyperspectral imaging system, which was composed of two hyperspectral cameras manufactured by SPECIM (Spectral Imaging Ltd., Oulu, Finland) to cover the ranges of visible and near-infrared (VNIR) and short-wavelength infrared (SWIR), a belt conveyor (Reall, Lublin, Poland) with belt speed regulated for each camera separately (to conduct line scanning of the plant leaves) and the illumination system Brilum (Piaseczno, Poland) model LAVADO416 with 4 lighting modules (each of them had 4x20W halogen lamps made by Philips, the Netherlands). The following imaging spectrographs were used inside the hyperspectral cameras: ImSpector V10E (400–1000 nm) and N25E 2/3″ imaging spectrometer (1000–2500 nm). The constant distance of 20 cm between the lenses of the cameras and the plant surfaces were maintained for each scan. The angle between the halogen lamps frames and the conveyor belt surface was 45°.

### Image acquisition and correction

The three-dimensional hyperspectral data, composed of 960 images of the plants (4 nutrient treatment × 5 development stages × 16 replications × 3 species of plants), were collected before chemometric analysis, and supervised classification were performed. Plants were scanned with the hyperspectral camera when staying in pots without uprooting. Data collection was performed five times during the experiment under differing stages of plant development, numbered as 1, 2, 3, 4 and 5. They represented 7, 14, 21, 35 and 49 days after transplanting (DAT) for celery plants; 7, 21, 31, 41 and 51 DAT for sugar beet plants; and 7, 14, 21, 35 and 45 DAT for strawberry plants. Description of development stages achieved by each plant species along with the code assigned on BBCH scale is provided in Table 4. For three studied species they covered the broad period from the unfolding of the second leaf till the appearance of the first fruit.

**Table 4:** The description of measurement dates based on BBCH (Biologische Bundesanstalt, Bundessortenamnt and Chemische Industrie) development stages [64, 65]

Measurement term	Plant species					
	Sugar beet		Celery		Strawberry	
	BBCH Code	Description	BBCH Code	Description	BBCH Code	Description
I	12	2nd true leaf unfolded	12	2nd true leaf unfolded	12	2nd true leaf unfolded
II	15	5th true leaf unfolded	14	4th true leaf unfolded	14	4th true leaf unfolded
III	19	9 or more true leaves unfolded	19	9 or more true leaves unfolded	58	Early balloon stage: first flowers with petals forming a hollow ball
IV	32	Leaves cover 20% of ground	42	20% of the expected root diameter reached	65	Full flowering: 50% of flowers open
V	35	Leaves cover 50% of ground	45	50% of the expected root diameter reached	85	First fruits have cultivar-specific colour

Hyperspectral images were recorded at a wavelength range of 400–1000 nm with a spectral resolution of 2.8 nm using a VNIR camera and in the range of 1000–2500 nm with a spectral resolution of 10 nm using a SWIR camera. During image acquisition, the plant samples were placed on the mobile platform using the scanning speed of 6 mm/s and 8 mm/s, and the camera exposure time was set to 2.3 and 7.6 ms for the VNIR and SWIR cameras, respectively. The hyperspectral images obtained during the measurements were recorded using data acquisition software SpectralDAQ ver. 2.1, which is specially dedicated to SPECIM cameras. For each series of measurements, white and dark calibrations were performed according to the procedure described in Baranowski et al. [24] to obtain the reflectance from the raw data.

### Hyperspectral image pretreatment

The corrected images were used to extract spectral information, select effective wavelengths, and elaborate the method of identification of P content in plants. First, the regions of interest (ROIs), including leaf surfaces, were pinpointed via ENVI software (ENVI5.4, Research System Inc., Boulder, CO, USA). Next, segmentation was implemented to segregate the ROIs. The segmentation was performed according to the procedure described by Baranowski et al. [59]. After image segmentation, reflectance values of all the pixels in each separate ROI were averaged to generate one mean value for each band. That way, the mean values of reflectance from 434 bands produced the representative reflectance spectrum of each sample. This procedure is referenced in the manuscript as the averaging of the reflectance spectra. Before the classification analysis, the extracted spectra were preprocessed using the second derivative, calculated with the Savitzky-Golay (SG) method (second-order polynomial and 11 smoothing points). The derivative processing suppresses the background signal as well as reduces image artefacts caused by non-uniform illumination [66]. Moreover, this pre-processing method increases the spectral resolution, has an input in the baseline correction, and enables an increased resolution of overlapping peaks [32]. Spectra obtained with the use of this preprocessing method are referenced in the manuscript as second derivative transformed spectra. The Unscrambler X ver. 10.1 (CAMO Software, Oslo, Norway) was used to pre-treat the spectral data.

### Effective wavelength selection

Hyperspectral image data contain large amounts of information, with redundancy and multi-collinearity between adjacent wavelengths, causing complex problems with their processing and application. To solve these problems, it is necessary to extract a number of essential wavelengths carrying the most relevant information before further analysis and implementation. To reduce the high dimensionality of the spectral data, the Correlation-Based Feature Selection (CFS) algorithm was applied to the SG transformed data. This algorithm uses heuristics that assign high scores to feature subsets that are highly correlated with the class and highly uncorrelated with each other [67]. In this research, a greedy-stepwise (GS) search strategy was applied in the CFS algorithm to select attributes through the space of subsets.

### Classification algorithms

Supervised classification experiments were performed on the hyperspectral data of the plant leaves. Different machine-learning algorithms, i.e., Backpropagation Neural Network (BNN), Random Forest (RF), Naive Bayes (NB) and Support Vector Machine (SVM), were used to classify plants under different phosphorus fertilizations at different developmental stages. Extensive work has been performed to optimize the parameters of individual models using trial and error. The parameters of the elaborated models are described in Table 5. For the classification experiment, samples were randomly selected for the test set and the validation set at the ratio 75:25. The training data set was used to build the classification model, while the test data set was used to check the model’s capability to properly classify new samples. The experiment of learning and testing was repeated 10 times with the random data selection (cross-validation method). All classification algorithms were implemented from a comprehensive software called the Waikato Environment for Knowledge Analysis, or WEKA [68]. Initially, the majority of available classifiers in these categories were tested on representative groups of training and test data. The four with the best prediction accuracies were chosen for comparison.

**Table 5:** Chosen features of the classifiers used in the study

Name of WEKA classifier’s library	Algorithm description	Acronym	Used parameters
Multilayer Perceptron	Neural networks with backpropagation used for tuning the weights of a neural net based on the error rate (i.e. loss).	BNN	AutoBuild: true; Learning rate: 0.3; Momentum: 0.1; Training time: 500 Hidden layers = 25
LibSVM	This library enables users to deal with One-class SVM, Regressing SVM, and nu-SVM. Many useful statistics are allowed including confusion matrix, precision estimation, ROC score.	LIBSVM	SVM Type: nu-SVC; Kernel Type: radial basis function; Nu: 0.g; gamma: 0.1; degree: 3 Normalize: true; Probability Estimates: true
Logistic	Used for building and using a multinomial logistic regression model with a ridge estimator.	LOG	Debug: false; MaxIts: −1; Ridge: 1.0E-6
Random Forests	This classifier enables to create forest of random trees. It induces each constituent decision tree from a bootstrap sample of the training data	RF	Debug: false; MaxDepth: 0; Num of Features: 0; Num of Trees: 10; Seed: 1

### Reference analysis

At the end of the experiment, fresh leaves from each plant were clipped from the canopy with a pair of scissors, put in plastic bags, frozen in a cooler and brought to the laboratory for measurements of leaf pigments and nutritional element content. To determine the chlorophyll content, fresh leaves (approximately 0.6 g) were ground in 80% acetone solution. Then, the leaf chlorophyll concentration was measured using a UV-VIS spectrophotometer (UV-5600, Metash, China) according to the method described by Lichtenthaler [69]. The rest of the plant samples were oven-dried (105 °C for 0.5 h followed by 80 °C until the constant weight was attained) and then ground into fine powder for mineral content analysis. The total P content was quantified spectrophotometrically using the vanado-molybdate phosphoric acid yellow colour method [70]. Total N content was determined by the micro Kjeldahls’ method. Total K concentration in the leaf was analysed using the flame-photometric method. The calcium content (Ca) and magnesium (Mg) content were determined using an atomic absorption spectrometer (Spektr AA 800, Varian, CA, USA).

### Statistical analyses

To test the effects of P fertilizer treatments on leaf photosynthetic pigments and nutritional elements, one-way analysis of variance (ANOVA) was followed by Tukey’s honest significant difference (HSD). *P*< 0.05 was considered statistically significant. Pearson’s correlation coefficient (R) was also used to test the relationship between leaf biochemical constituents, P fertilization and parts of the plants. All these statistical analyses were conducted using STATISTICA v13.4 (TIBCO Software Inc., Palo Alto, California, United States).

## Supplementary Information

**Additional file 1: Table S1.** Confusion matrices created for random forest (RF) models of phosphorous content in the treatments for three studied species (sugar beet – in blue, celery – in green and strawberry – in red) at five stages of plant growth.

## Data Availability

The datasets generated during the current study are available from the corresponding author on reasonable request after consulting the project funder.

## Abbreviations

VNIR: Visible and near infrared
PCC: Pearson correlation coefficients
ROI: Regions of interest
SWIR: Short-wave infrared
CFS: Correlation-based feature selection
NIR: Near infrared
DAT: Days after transplanting
SG: Savitzky-Golay
GS: Greedy-stepwise
UV-VIS: Ultraviolet-visible
BNN: Backpropagation neural network
RF: Random forest
NB: Naive Bayes
SVM: Support vector machine
WEKA: Waikato environment for knowledge analysis
ANOVA: Analysis of variance
HSD: Honest significant difference
