# Supplementary material for: Identification of plant leaf phosphorus content at different growth stages based on hyperspectral reflectance
Source: BMC Plant Biol. 2021 Jan 7;21:28. doi: 10.1186/s12870-020-02807-4 (PMC7792193; doi:10.1186/s12870-020-02807-4)
Supplement: Supplementary file 1 — Additional file 1: Table S1. Confusion matrices created for random forest (RF) models of phosphorous content in the treatments for three studied species (sugar beet – in blue, celery – in green and strawberry – in red) at five stages of plant growth. [file 12870_2020_2807_MOESM1_ESM.docx]

**SUPPLEMENT 1**

Table S1. Confusion matrices created for random forest (RF) models of phosphorous content in the treatments for three studied species (sugar beet – in blue, celery – in green and strawberry – in red) at five stages of plant growth

| I | | | | | | | | | | | | |
| --- | --- | --- | --- | --- | --- | --- | --- | --- | --- | --- | --- | --- |
|  | **P-33** | | | **P-67** | | | **P-100** | | | **P-133** | | |
| P-33 | 60% | 100% | 80% | 40% | 0% | 0% | 0% | 0% | 20% | 0% | 0% | 0% |
| P-67 | 0% | 0% | 0% | 100% | 100% | 20% | 0% | 0% | 80% | 0% | 0% | 0% |
| P-100 | 0% | 0% | 0% | 40% | 0% | 20% | 60% | 100% | 80% | 0% | 0% | 0% |
| P-133 | 20% | 20% | 0% | 40% | 60% | 0% | 0% | 0% | 0% | 40% | 20% | 80% |
| II | | | | | | | | | | | | |
|  | **P-33** | | | **P-67** | | | **P-100** | | | **P-133** | | |
| P-33 | 80% | 40% | 100% | 0% | 40% | 0% | 20% | 20% | 0% | 0% | 0% | 0% |
| P-67 | 0% | 40% | 0% | 60% | 20% | 100% | 40% | 40% | 0% | 0% | 0% | 0% |
| P-100 | 0% | 20% | 0% | 20% | 20% | 20% | 80% | 60% | 80% | 0% | 0% | 0% |
| P-133 | 20% | 0% | 0% | 20% | 0% | 0% | 20% | 0% | 0% | 60% | 100% | 100% |
| III | | | | | | | | | | | | |
|  | **P-33** | | | **P-67** | | | **P-100** | | | **P-133** | | |
| P-33 | 100% | 100% | 100% | 0% | 0% | 0% | 0% | 0% | 0% | 0% | 0% | 0% |
| P-67 | 0% | 0% | 0% | 60% | 100% | 100% | 40% | 0% | 0% | 0% | 0% | 0% |
| P-100 | 0% | 0% | 0% | 60% | 60% | 20% | 40% | 40% | 80% | 0% | 0% | 0% |
| P-133 | 0% | 0% | 0% | 0% | 0% | 0% | 20% | 20% | 0% | 80% | 80% | 100% |
| IV | | | | | | | | | | | | |
|  | **P-33** | | | **P-67** | | | **P-100** | | | **P-133** | | |
| P-33 | 80% | 80% | 100% | 0% | 20% | 0% | 0% | 0% | 0% | 20% | 0% | 0% |
| P-67 | 0% | 0% | 0% | 100% | 100% | 100% | 0% | 0% | 0% | 0% | 0% | 0% |
| P-100 | 0% | 0% | 0% | 0% | 0% | 0% | 60% | 80% | 100% | 40% | 20% | 0% |
| P-133 | 40% | 0% | 0% | 0% | 0% | 0% | 0% | 20% | 0% | 60% | 80% | 100% |
| V | | | | | | | | | | | | |
|  | **P-33** | | | **P-67** | | | **P-100** | | | **P-133** | | |
| P-33 | 80% | 80% | 80% | 0% | 20% | 20% | 20% | 0% | 0% | 0% | 0% | 0% |
| P-67 | 0% | 0% | 0% | 100% | 100% | 80% | 0% | 0% | 20% | 0% | 0% | 0% |
| P-100 | 0% | 0% | 0% | 20% | 0% | 20% | 80% | 100% | 80% | 0% | 0% | 0% |
| P-133 | 0% | 0% | 20% | 0% | 0% | 0% | 0% | 0% | 0% | 100% | 100% | 80% |
